# Dioxin Exposure, from Infancy through Puberty, Produces Endocrine Disruption and Affects Human Semen Quality

**DOI:** 10.1289/ehp.10399

**Published:** 2007-10-29

**Authors:** Paolo Mocarelli, Pier Mario Gerthoux, Donald G. Patterson, Silvano Milani, Giuseppe Limonta, Maria Bertona, Stefano Signorini, Pierluigi Tramacere, Laura Colombo, Carla Crespi, Paolo Brambilla, Cecilia Sarto, Vittorio Carreri, Eric J. Sampson, Wayman E. Turner, Larry L. Needham

**Affiliations:** 1 University Department of Laboratory Medicine, Hospital of Desio, Milano, Italy; 2 School of Medicine, University Milano-Bicocca, Milano, Italy; 3 National Center for Environmental Health, Centers for Disease Control and Prevention, Atlanta, Georgia, USA; 4 Institute of Medical Statistics and Biometrics, University of Milano, Milano, Italy; 5 Department for Preventive Medicine, Ministry of Health of Regione Lombardia, Milano, Italy

**Keywords:** dioxin, endocrine disruption, environmental contaminants, human sperm quality, reproductive hormones, TCDD

## Abstract

**Background:**

Environmental toxicants are allegedly involved in decreasing semen quality in recent decades; however, definitive proof is not yet available. In 1976 an accident exposed residents in Seveso, Italy, to 2,3,7,8-tetrachlorodibenzo-*p*-dioxin (TCDD).

**Objective:**

The purpose of this study was to investigate reproductive hormones and sperm quality in exposed males.

**Methods:**

We studied 135 males exposed to TCDD at three age groups, infancy/prepuberty (1–9 years), puberty (10–17 years), and adulthood (18–26 years), and 184 healthy male comparisons using 1976 serum TCDD levels and semen quality and reproductive hormones from samples collected 22 years later.

**Results:**

Relative to comparisons, 71 men (mean age at exposure, 6.2 years; median serum TCDD, 210 ppt) at 22–31 years of age showed reductions in sperm concentration (53.6 vs. 72.5 million/mL; *p* = 0.025); percent progressive motility (33.2% vs. 40.8%; *p* < 0.001); total motile sperm count (44.2 vs. 77.5 × 10^6^; *p* = 0.018); estradiol (76.2 vs. 95.9 pmol/L; *p* = 0.001); and an increase in follicle-stimulating hormone (FSH; 3.58 vs. 2.98 IU/L; *p* = 0.055). Forty-four men (mean age at exposure, 13.2 years; median serum TCDD, 164 ppt) at 32–39 years of age showed increased total sperm count (272 vs. 191.9 × 10^6^; *p* = 0.042), total motile sperm count (105 vs. 64.9 ×10^6^; *p* = 0.036), FSH (4.1 vs. 3.2 UI/L; *p* = 0.038), and reduced estradiol (74.4 vs. 92.9 pmol/L; *p* < 0.001). No effects were observed in 20 men, 40–47 years of age, who were exposed to TCDD (median, 123 ppt) as adults (mean age at exposure, 21.5 years).

**Conclusions:**

Exposure to TCDD in infancy reduces sperm concentration and motility, and an opposite effect is seen with exposure during puberty. Exposure in either period leads to permanent reduction of estradiol and increased FSH. These effects are permanent and occur at TCDD concentrations < 68 ppt, which is within one order of magnitude of those in the industrialized world in the 1970s and 1980s and may be responsible at least in part for the reported decrease in sperm quality, especially in younger men.

In the last 50 years a significant global decline in human sperm concentrations of about 1% per year (Auger et al. 1995; Carlsen et al. 1992; Menchini-Fabris et al. 1996; Sharpe and Skakkebaek 1993; Swan et al. 2000) has been reported in Western countries, although with regional differences (Jorgensen et al. 2001; Swan et al. 2003). Furthermore, the youngest generations within a single country have been found to have lower sperm counts (Andersen et al. 2000; Auger et al. 1995; Van Waeleghem et al. 1996).

These phenomena may be related to increasing exposures to estrogenic, anti-estrogenic, or antiandrogenic chemicals during critical phases of testicular development (Damstra et al. 2002; Sharpe 2001; Sharpe and Skakkebaek 1993). Exposures to poly-chlorinated dibenzo-*p*-dioxins (PCDDs), polychlorinated biphenyls (PCBs), and poly-chlorinated dibenzofurans (PCDFs), which are products and by-products of industrial or combustion processes, have the potential to disrupt multiple endocrine pathways and induce toxic responses. For example, experimental animal data have shown adverse effects in testicular function, including reduced sperm counts and motility, after exposure to 2,3,7,8-tetrachlorodibenzo-*p*-dioxin (TCDD) (Faqi et al. 1998; Mably et al. 1992; Roman and Peterson 1998).

The prenatal and perinatal periods are particularly sensitive, and indeed, higher exposure doses are required to produce similar effects in adult animals (Damstra et al. 2002; Roman and Peterson 1998; Theobald et al. 2003). No definitive data are available for men, but Guo et al. (2000) observed alterations in sperm morphology and motility after prenatal exposure to PCBs/PCDFs in the Yucheng cohort, and Hauser et al. (2005) reported a decrease in sperm motility as a consequence of exposure to PCBs and phthalates in adults.

An explosion on 10 July 1976 at a trichlorophenol manufacturing plant near Seveso, Italy (Bertazzi et al. 2001; Mocarelli et al. 1986) released up to 30 kg of TCDD (Di Domenico et al. 1990; Needham et al. 1997).

We investigated the relationship between serum TCDD concentrations in 1976 and semen quality and male reproductive hormones 22 years later. The men studied were exposed either during infancy/prepuberty, puberty, or during early adult life.

## Materials and Methods

### Participants

**Figure 1:**
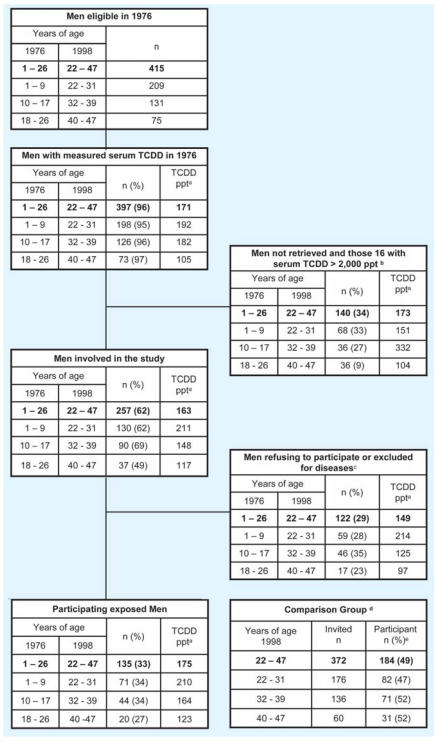
Flow chart of study showing the relationship between eligible men in 1976, participant men in 1998, and the comparison group on the effects of exposure to TCDD at different ages (1–9; 10–17; 18–26 years) on reproductive hormones and semen quality. Values in parentheses indicate the percentage of men respective to the eligible ones. ***^a^***Median TCDD serum concentration in 1976 (ppt on a serum lipid basis). ***^b^***Very highly exposed men (> 2,000 ppt) were excluded: 10 men who were 1–9 years old in 1976 and 6 men who were 10–17 years old in 1976, with median serum concentrations of 6,350 ppt and 3,700 ppt, respectively; none of the men exposed at 18–26 years of age was exposed to > 2,000 ppt TCDD. ***^c^***For information about this group, see Table 1. ***^d^***Serum TCDD concentrations for the comparison groups were assumed to be ≤ 15 ppt in 1976 and < 6 ppt in 1998. ***^e^***Values in parentheses indicate compliance of the comparison group.

A total of 397 Caucasian males (of the eligible 415) from the highly TCDD-contaminated A zone (Di Domenico et al. 1990; Needham et al. 1997) and from nearby contaminated areas, all of whom were 1–26 years of age in 1976, were invited to participate in the study conducted in 1997–1998 (Figure 1). Frozen serum samples (generally ≤ 1 mL in volume) from blood collected in 1976–1977 from these subjects were available for TCDD measurements.

A total of 372 consecutive healthy volunteer blood donors the same age as the exposed men, but not living in TCDD-contaminated areas (i.e., they were not exposed to TCDD by the Seveso explosion) were also invited to participate (Figure 1). All participants were first screened for any hidden disease by clinical laboratory tests for liver, bone marrow, kidney, and pancreatic functions. All participants with specific diseases or conditions (Table 1) were excluded.

The participants completed a questionnaire on health and socioeconomic status and donated blood and semen samples (samples were collected the morning after having been sexually abstinent for at least 3 days). The study protocol was approved by the Institutional Human Subjects Committee. All study participants gave written informed consent.

### Laboratory data

#### Semen samples

Participants collected a postmasturbatory semen sample at home. Each sample was transported at approximately body temperature to the Desio Hospital laboratory and kept at 37°C until examination, which occurred within 1 hr after ejaculation and tests were performed in blind by the same two technicians according to the World Health Organization (WHO 1982) recommendation. Ejaculate volume was calculated gravimetrically. Sperm motility was assessed at 400× magnification on a microscope heating stage (37°C) in duplicate, and the average value was recorded. Sperm concentration was measured using a Bürker-Türk chamber at phase contrast (400× magnification). Morphology was evaluated by the same observer on 300 Papanicolaou-stained sperm per slide (David et al. 1975; Jouannet et al. 1988).

#### Serum hormone analyses

Fasting blood samples were obtained on the same morning as semen collection. An aliquot of serum was stored at −80°C and analyzed for hormone levels in large batches to reduce interassay variability. Serum 17β-estradiol (E_2_), follicle-stimulating hormone (FSH), inhibin B, and luteinizing hormone (LH) were measured according to established immunofluorimetric methods, and testosterone was measured by radioimmunoassay. Quality control protocols were applied with strict criteria for all tests.

#### Serum TCDD measurements

Vials containing 0.6–1.0 mL serum samples stored frozen since 1976–1977 were analyzed for TCDD by isotope-dilution mass spectrometry at the Centers for Disease Control and Prevention (Patterson et al. 1987). Serum TCDD concentrations (parts per trillion on serum lipid basis) were also determined in samples drawn in 1997–1998 from all individuals whose 1976–1977 serum TCDD value exceeded 15 ppt [then the “background level” (Needham et al. 1997)] and in pooled samples of men from uncontaminated areas to assess the background levels in 1998/2002. The samples with concentrations less than the detection limit were assigned a value half of that limit.

### Statistical analyses

We established and maintained a general database using SAS software (version 8.2; SAS Institute Inc., Cary, NC, USA). The exposed and comparison groups were divided, according to the developmental stage of the reproductive system (Sharpe et al. 2003), into three 1976 age classes: infancy/prepuberty, puberty, and young adult (1–9, 10–17, and 18–26 years of age, respectively). Sensitivity analyses were performed to test the cutoff among the age groups. Sperm and hormone data were fitted with a general linear model including group, age class, interaction group × age class as terms, and with abstinence length (not considered for hormone analysis), smoking status (total number of cigarettes smoked per day during months of habitual smoking), body mass index (BMI), and chronic exposure to solvents and other toxic substances in the furniture-manufacturing industry as covariates. We applied scale transformations to approximate normal distribution and homoscedasticity: sperm concentration, total sperm count, progressive motile sperm count, and concentrations of E_2_, testosterone, and FSH were log-transformed; semen volume and concentrations of LH and inhibin B were square-root–transformed. Results were expressed as back transformation of least squares means (i.e., the means adjusted for all the terms in the model). Two families of comparisons were considered: “among groups within age-class” and “among age classes within group.” According to the Bonferroni principle, a 0.025 comparison-wise risk of type I error ensures a family-wise risk of type I error ≤ 0.05.

## Results

The biological and socioeconomic characteristics of the two study groups were similar, except for a higher education level and lower occupation in the furniture-manufacturing industry in the comparison group (Table 1). These differences did not affect the comparison between exposed and unexposed groups; the effect of the inclusion of these variables as covariates in the model was negligible.

The incidence of self-reported varicocele or cryptorchidism were not statistically different in the exposed and comparison groups; however, we excluded these men from the analyses.

**Figure 2:**
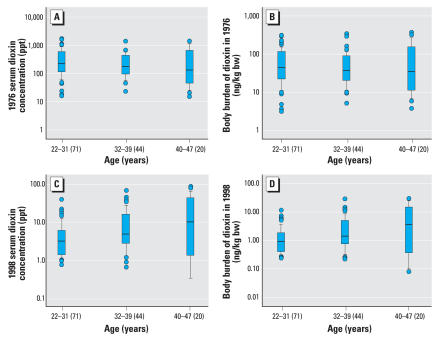
Box plots showing dioxin concentration on a serum lipid basis (*A,C*) and body burden [ng/kg body weight (bw); *B,D*] in the same men in 1976 (*A, B*) and in 1998 (*C, D*). Values shown are median (line within box), 25th and 75th percentiles (bottom and top of box, respectively), and outliers (circles). Whiskers indicate values within 1.5 times the interquartile range (25th–75th percentiles), and values in parentheses indicate number of men. Serum dioxin concentrations in comparison groups were < 15 ppt in 1976 and < 6.0 ppt in 1998. Because weight was not available in medical records for most of the subjects, dioxin body burden was mostly derived in 1976 using normal percentile distribution of weight according to age.

The 1976 serum TCDD concentrations of eligible men who did and did not participate in the study were similar (Figure 1). The TCDD concentrations (Figure 2A, 2B) were also comparable among exposed age groups. Median serum TCDD levels in 1998 (Figure 2C, 2D) were higher in males exposed in 1976 as adults than in males who were exposed as children. This is in agreement with the much shorter TCDD half-life in children (Aylward et al. 2005; Kreuzer et al. 1997), but this observation did not explain any statistically significant effects.

We assumed that serum TCDD concentrations for the comparison groups were ≤ 15 ppt in 1976–1977 (Needham et al. 1997) and < 6 ppt in 1998/2002 on the basis of serum results for residents of uncontaminated areas around Seveso (Mocarelli P, unpublished data). Because the only dioxin-like chemical involved with the Seveso incident was TCDD, we focused on TCDD for these analyses. If TCDD acts in concert with other dioxin-like chemicals in affecting sperm quality, the total dioxin toxic equivalency (TEQ) should be considered. In nine serum pools from females residing in the uncontaminated area in 1976, Eskenazi et al. (2004) found an average TEQ of 100 ppt.

### TCDD exposure and semen quality

**Figure 3:**
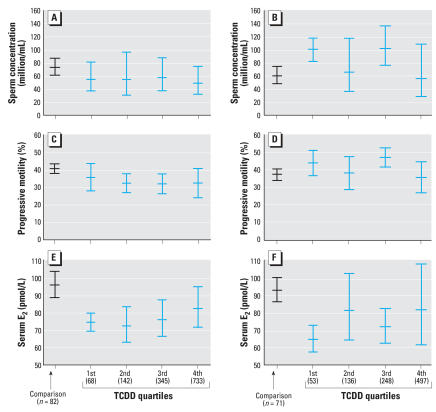
TCDD quartile distribution (adjusted mean and 95% confidence interval) of sperm concentration (*A, B*), total motile sperm count (*C, D*), and serum E_2_ (*E, F*) for exposed men and of same-age comparison groups [*A,C,E*; men who were 1–9 years of age in 1976 (22–31 years of age in 1998); *B,D,F*; men who were 10–17 years of age in 1976 (32–39 years of age in 1998). Median concentrations of TCDD quartiles (shown in parentheses) are expressed as parts per trillion on a serum lipid basis in 1976.

In 71 men exposed at 1–9 years of age (mean, 6.2 years), serum TCDD concentrations (median, 210 ppt) had a significant effect on semen quality measured 22 years later. Indeed, significant decreases in sperm count (*p* = 0.025), progressive sperm motility (*p* = 0.001), and total number of motile sperm (*p* = 0.01) were observed relative to the comparison group (Table 2). Quartile distribution (Figure 3A, 3C) shows that serum TCDD concentrations ≤ 113 ppt (median of first quartile, 68 ppt) adversely affected sperm concentration and total motile sperm count.

In contrast to the observed effects on men exposed at 1–9 years of age, exposure (median TCDD serum concentration, 164 ppt) at 10–17 years of age (mean, 13.2 years) resulted in effects that appeared to be stimulatory to semen parameters (Table 2 and Figure 3B, 3D).

In 20 men 40–47 years of age who were exposed to TCDD (serum concentration, 15.5–1,310 ppt; median, 123 ppt) at 18–26 years of age (mean, 21.5 years), we found no statistically significant differences for any of the sperm variables compared with the 32 men in the comparison group. Moreover, no trends in these variables were related to different TCDD serum concentrations in 1998. Also, we observed no statistically significant differences for sperm morphology between exposed and comparison groups.

### TCDD exposure and hormone levels

Men exposed to TCDD at 1–9 and 10–17 years of age had lower serum E_2_ concentrations (*p* < 0.001) and higher serum FSH concentrations (*p* = 0.055 and *p* = 0.038, respectively) than the comparison groups (Table 2). We found differences in E_2_ at TCDD concentrations < 53 ppt (Figure 3E, 3F). In contrast, subjects exposed at 18–26 years of age (mean, 21.5 years) showed no differences in concentrations of E_2_ (*p* = 0.248) or other hormones relative to the comparison group. Exposure status had no effect on testosterone or inhibin B concentrations in any group.

### Comparison between TCDD exposure during infancy and during puberty

The semen of men exposed to TCDD at 1–9 years of age presented significantly greater effects relative to their respective comparisons than semen from men exposed at 10–17 years of age (Table 2). Indeed, the former group showed statistically significant lower sperm concentrations (*p* = 0.008), total sperm counts (*p* = 0.004), progressive motility (*p* = 0.005), and total motile sperm counts (*p* < 0.001) than the latter. No statistically significant differences were noted among the comparison groups (Table 2).

## Discussion

This study on men from Seveso provides evidence of a permanent disruptive effect of TCDD on the human male reproductive system, depending on the age at exposure. Prepubertal children (< 9 years of age) are very sensitive to TCDD, with a reduction of sperm concentration and motility observed at serum levels < 68 ppt (equivalent to a body burden of about 12 ng/kg body weight). In contrast, exposure to TCDD during puberty causes an increase of these semen parameters. If men who are first exposed at 1–9 years of age continued to be exposed at 10–17 years of age to higher than background levels, we would expect the effects to balance out. However, this is not the case. One possible explanation is the much shorter TCDD half-life (months, not years) in young children. Therefore, some of the children exposed at 1–9 years of age may have had a low dose of TCDD (> 15 ppt background level) still present at puberty, which did not determine a stimulatory effect; the other possibility is the presence of a higher dose at puberty, which nevertheless did not produce a stimulatory effect. Therefore, this contributes to the hypothesis of a permanent effect (Mocarelli et al. 2000); note the striking differences between men exposed at 1–9 years of age compared with those exposed at 10–17 years as shown in Table 2. Indeed, exposure to endocrine-disrupting chemicals during the period when “programming” of the endocrine system is in progress may result in a permanent change of function or sensitivity to stimulatory/inhibitory signals (Damstra et al. 2002). One consequence of these opposite effects in infancy compared with puberty could be that the action of dioxin and similar pollutants in the general male population is obscured because the two effects could cancel out each other to give an average normal appearance.

However, in both age groups, TCDD exposure results in a significant reduction in serum E_2_ levels in adulthood. It is important to note that the TCDD body burden and serum levels of these men were within background levels for that time period, demonstrating a permanent effect of the original low dose they received (Figure 2). No effect was observed at all when exposure to TCDD occurred during adulthood.

This study has several strengths. First, we have clearly and directly related original exposure levels of the ubiquitous environmental endocrine disruptor TCDD to reproductive outcomes years after exposure. Second, the participants of the study are fully representative of the available eligible population. Third, the TCDD concentrations affecting children, particularly boys, is similar to the maternal TCDD body burden that has been shown to induce a reduction in sperm numbers in adult rats exposed *in utero* and/or during lactation to TCDD (Faqi et al. 1998; Mably et al. 1992; Roman and Peterson 1998; Theobald et al. 2003) and to serum concentration shown to decrease the sex ratio (Mocarelli et al. 1996 and 2000) in offspring of TCDD-exposed men at Seveso. These serum concentrations are also lower than the concentrations shown at Seveso to induce a slight, nonstatistically significant increased risk of endometriosis (Eskenazi et al. 2002) and breast cancer (Warner et al. 2002) in women, and developmental dental aberrations in men exposed at ages younger than 5 years (Alaluusua et al. 2004).

However, the study may be weak because of sampling problems involving voluntary sperm analysis. A bias, mainly due to low compliance (∼ 20–40%), has been recorded using sampling as a representation of the general population (Jorgensen et al. 2001). To deal with such bias, we chose healthy blood donors from a nearby area; this group of men showed a high compliance (∼ 60%) (Table 1) and may be considered representative of the general healthy male population. In any case, we were able to overcome a possible bias by the observation of very significant differences between the 22- to 31-year-old and the 32- to 39-year-old exposed groups (Table 2), whereas no differences were seen between the equivalent comparison groups.

A possible role of chronic exposure to solvents or other toxic substances used in the furniture-manufacturing industry has been ruled out by similar exposure (Table 1) and by multivariate statistical analysis.

Currently, no data directly relate TCDD exposure at a young age with human sperm quality. The only similar data are those on effects of PCBs and phthalates: Hauser et al. (2005) reported a decrease of sperm motility after exposure to PCBs and phthalates as in our case; and Guo et al. (2000) observed alterations in sperm morphology after prenatal exposure to PCBs/PCDFs (unlike our data) after an incident in Taiwan in 1979. Also, in Taiwan, men exposed to PCBs/PCDFs at 18–30 years of age showed abnormal sperm morphology (Huang et al. 2003). These researchers, however, did not measure PCB/PCDF concentrations at exposure and did not show modification in sperm number, as is the case in the present study. This effect, present in experimental animals (Roman and Peterson 1998; Theobald et al. 2003), could have gone unnoticed because of the absence of exposure data on children in those studies.

### Possible explanations

The contrasting effects of infant versus pubertal TCDD exposure on sperm count and the lack of effect in adults may have a physiologic explanation related to differences in the hormonal regulation of Sertoli cell proliferation with age (Sharpe et al. 2003).

Final Sertoli cell number is the main determinant (other than abstinence period) of sperm count in men (Sharpe et al. 2003). Proliferation of these cells in humans occurs during three periods: fetal, postnatal (0–8 months of age), and probably prepubertal. Thus, in the present study, a similar exposure during the prepubertal period (average age, 6.2 years) suppresses Sertoli cell number, whereas exposure during the peripubertal period stimulates Sertoli cell number. This differential action may reflect diversities in the mechanisms that regulate Sertoli cell proliferation at these two time points. Androgens may be the primary stimulator of perinatal and prepubertal proliferation (Atanassova et al. 2005), whereas peripubertal proliferation is driven principally by FSH (Johnston et al. 2004). Differential effects of TCDD on androgen and FSH action in infancy compared with puberty may provide a ready explanation for the observed differences in sperm count.

TCDD and other dioxin-like chemicals produce their effects primarily through the aryl hydrocarbon receptor (AhR). Activation of AhR by dioxin, therefore, could be a mechanism by which androgen action is reduced; this could explain the observed decrease in sperm count in adults who were exposed to TCDD as young children (i.e., when Sertoli cell development is more testosterone dependent). This hypothesis is supported by the observation that *in utero* exposure of human males to maternal smoking causes reduced sperm counts in the offspring at adulthood; this probably is a result of reduced Sertoli cell number (Jensen et al. 2004; Storgaard et al. 2003) due to the action of polycyclic aromatic hydrocarbons present in cigarette smoke on AhR.

In contrast, when TCDD contamination occurs at puberty, Sertoli cell proliferation is primarily FSH dependent. E_2_ is a potent negative regulator of FSH secretion, and studies have shown that E_2_ suppression of FSH can reduce Sertoli cell proliferation and number (Johnston et al. 2004).

TCDD-induced reduction of E_2_ levels (and corresponding elevation of FSH levels), as shown in adults exposed during infancy or puberty (present study), may indicate that increased FSH levels during puberty may lead to increased Sertoli cell proliferation, and hence, to higher sperm counts in adulthood. Although a similar change may have occurred in boys exposed during infancy, the effect of FSH on Sertoli cell proliferation at this age may be insignificant and/or it may be counteracted by the negative repercussions related to suppression of androgen action. Exposure to TCDD after puberty (i.e., after completion of the reproductive system) would not modify estrogen concentration or semen quality, which is consistent with our results.

Taken together, our data are consistent with the untested hypothesis that TCDD exposure during sensitive developmental “windows” may affect expression of responsive genes (with or without the effects of estrogens and/or androgens), permanently altering the programming of the primordial germ cells.

The effect of AhR signaling could be stimulatory or inhibitory, depending on the interplay of factors that include the level of dioxin exposure, the period of sensitivity and/or development of the target cells, and the actual level of key regulatory molecules, including the androgen-estrogen balance. It could also explain the lack of effect of TCDD on spermatogenesis of the mature reproductive system and the “normal morphology” of sperm of exposed men.

## Conclusions

Our results directly demonstrate a reduction in E_2_ and a permanent effect on semen quality in human males as a result of the disruptive action of low concentrations of TCDD on the endocrine system. This occurs after exposure especially in infancy/prepuberty, less in puberty, and not in adulthood, at levels, until recently, that were seen in the general population of many industrialized countries. Our data could explain, at least in part, the reported reduction (Andersen et al. 2000; Menchini-Fabris et al. 1996; Van Waeleghem et al. 1996) of semen quality of the youngest populations in Western countries. In fact, these data demonstrate that serum concentrations of about 100 TEQ are border limits; however, at these levels, effects on E_2_ concentration and on the developing male reproductive system begin to be produced. Certain human populations, especially children during breast-feeding (Link et al. 2005), may have a total body burden of dioxin-like chemicals close to this limit. Sensitive children can also be affected at lower concentrations; it will be of interest to see if, as a result of public health efforts in decreasing dioxin levels [from a TEQ level in children in Seveso in 1976 of about 100 ppt (Eskenazi et al. 2004) to about 10 ppt in Germany in 2002/2003 (Link et al. 2005)], there will be a reversal in the reported reduction of semen quality. One remaining significant question will be to determine whether *in utero* exposure will affect human sperm quality.

## Figures and Tables

**Table 1:** Characteristics of study participants by age at time of study.

	Exposed (*n* = 257)			Comparison (*n* = 372)		
Characteristic by age group	22–31	32–39	40–47	22–31	32–39	40–47
Age class (years)						
At exposure to dioxin in 1976	1–9	10–17	18–26			
No. recruited for the study	130	90	37	176	136	60
No. refused	40	29	11	75	52	23
No. interested	90	61	26	101	84	37
Percent compliance	69	68	70	57	62	62
No. excluded from the study (*n*)^a^						
Diabetes	—	1	2	—	—	—
Epididymitis	1	—	—	1	1	—
Urological diseases	1	1	—	1	—	1
Vasectomy	—	—	2	—	—	—
Hormonal therapy	1	1	—	1	—	—
Hyperthyroidism	—	—	—	—	—	1
Varicocele	9	6	1	9	8	3
Cryptorchidism	7	6	0	6	2	1
Varicocele and cryptorchidism	0	2	1	1	2	0
Participants (*n*)	71	44	20	82	71	31
Age [years (mean ± SD)]						
At the time of the test	28.1 ± 2.5	35.0 ± 2.2	43.3 ± 2.2	27.3 ± 2.9	35.5 ± 2.3	43.1 ± 2.3
At exposure to dioxin in 1976	6.2 ± 2.5	13.2 ± 2.2	21.5 ± 2.2			
BMI (%)^b^						
< 25	53.5	54.5	45.0	76.8	53.5	41.9
25–30	45.1	40.9	40.0	20.7	39.4	48.4
> 30	1.4	4.6	15.0	2.5	7.1	9.7
Education level (%)						
≤ Middle school	46.5	61.4	70.0	29.3	39.4	51.6
High school	46.5	34.1	25.0	42.6	43.7	35.5
University	4.2	4.5	5.0	11.0	16.9	12.9
Current student	2.8	—	—	17.1	—	—
Tobacco use (%)						
Never	47.9	40.9	40.0	46.3	43.7	32.3
Former	25.4	29.5	45.0	19.5	23.9	45.2
Current						
≤ 5 cigarettes/day	1.4	9.1	5.0	3.7	—	—
> 5 cigarettes/day	25.3	20.5	10.0	30.5	32.4	22.5
Maternal smoking during pregnancy (%)						
No	77.4	81.8	90.0	86.6	81.7	87.1
Yes	11.3	13.6	10.0	8.5	16.9	—
Unknown	11.3	4.6	—	4.9	1.4	12.9
Alcohol use (%)						
≤ 10 g /day	18.3	15.9	20.0	22.0	31.0	22.6
11–20 g /day	2.8	4.5	—	8.5	5.6	3.2
21–30 g /day	8.5	6.9	—	6.1	1.4	9.7
> 30 g /day	70.4	72.7	80.0	63.4	62.0	64.5
Employment status (%)						
Industry						
Furniture manufacturing	10.0	38.6	20.0	7.3	4.2	6.5
Other	45.0	18.2	10.0	24.4	18.3	19.4
Clerks	23.9	15.9	25.0	35.4	28.2	35.5
School, other	21.1	27.3	45.0	32.9	49.3	38.6
Exposure to chemical substances (%)^c^	69.0	82.1	80.0	42.7	49.3	38.7

a Excluded for self-reported causes (questionnaire) or because of pathologic results of clinical laboratory tests (aspartate aminotransferase, alanine aminotransferase, gamma-glutamyl transferase, total cholesterol, high-density lipoprotein cholesterol, low-density lipoprotein cholesterol, C-reactive protein, glucose, creatinine, complete blood cell count and differential, hemoglobin, hepatitis B surface antigen, hepatitis B core antibody, hepatitis C. Urine analysis was performed for all subjects.

b kg/m^2^.

c Mostly organic solvents, adhesives, paints, colors, and powders (wood, hides, metals).

**Table 2:** Differences in sperm and hormone data between men exposed to TCDD and nonexposed comparison groups by age at time of study (age at dioxin exposure in 1976).

					*p-*Value^a^			
	EG		CG		EG vs. CG		22–31 vs. 32–39	
Characteristic by age group	22–31 (1–9)	32–39 (10–17)	22–31	32–39	22–31	32–39	22–31	32–39
Participants (*n*)	71	44	82	71				
TCDD exposure (ppt^b^ level median)								
In 1976	210^c^	164^c^	≤15^d^	≤15^d^				
In 1998	3.04	4.67	< 6.0	< 6.0				
Sperm concentration (10^6^/mL)					0.025	0.213	0.008	0.817
Mean^e^	53.6	81.9	72.5	60.8				
Mean ± SD^e^	21.8–131.8	37.8–177.9	31.7–165.9	24.2–152.8				
Adjusted mean^e^	48.6	87.4	67.1	70.5				
Adjusted mean ± SE	43.1–54.8	74.7–102.3	59.4–75.7	61.3–81.1				
Total sperm count (10^6^)					0.168	0.042	0.004	0.663
Mean	154.6	272.0	204.4	191.9				
Mean ± SD	55.8–428.2	132.3–559.0	83.9–498.1	62.8–586.0				
Adjusted mean	149.8	302.8	186.1	206.5				
Adjusted mean ± SE	131.5–170.7	255.2–359.3	163.2–212.3	177.3–240.4				
Semen volume (mL)					0.196	0.179	0.662	0.818
Mean	3.17	3.48	3.06	3.39				
Mean ± SD	1.50–5.46	2.12–5.17	1.64–4.93	1.87–5.36				
Adjusted mean	3.40	3.60	3.03	3.13				
Adjusted mean ± SE	3.16–3.65	3.28–3.93	2.81–3.27	2.86–3.41				
Progressive motility (%)^f^					0.001	0.187	0.005	0.673
Mean	33.2	41.1	40.8	37.4				
Mean ± SD	19.6–46.9	28.9–53.3	27.3–54.2	23.4–51.3				
Adjusted mean	32.4	42.1	40.0	38.5				
Adjusted mean ± SE	30.5–34.3	39.6–44.6	38.1–41.9	36.3–40.7				
Total motile sperm count (10^6^)^g^					0.018	0.036	< 0.001	0.866
Mean	44.2	105.2	77.5	64.9				
Mean ± SD	11.2–174.0	40.0–277.0	25.6–234.5	15.8–267.0				
Adjusted mean	41.8	121.9	68.4	72.2				
Adjusted mean ± SE	35.2–49.7	97.2–152.7	57.5–81.5	59.1–88.3				
E_2_ (pmol/L)					0.001	0.001	0.350	0.240
Mean	76.2	74.4	95.9	92.9				
Mean ± SD	59.4–97.7	54.5–101.6	67.1–137.2	67.8–127.3				
Adjusted mean	73.5	79.3	90.3	99.3				
Adjusted mean ± SE	70.4–76.8	74.8–83.9	86.4–94.4	94.3–104.5				
FSH (IU/L)					0.055	0.038	0.384	0.600
Mean	3.58	4.10	2.98	3.20				
Mean ± SD	2.03–6.32	2.84–5.93	1.68–5.28	1.69–6.05				
Adjusted mean	3.58	4.06	2.98	3.22				
Adjusted mean ± SE	3.31–3.87	3.66–4.51	2.75–3.23	2.93–3.53				
Testosterone (nmol/L)					0.493	0.145	0.515	0.981
Mean	16.4	14.3	17.7	15.2				
Mean ± SD	12.4–21.8	10.7–19.0	13.6–23.1	11.8–19.5				
Adjusted mean	15.9	15.3	16.4	16.4				
Adjusted mean ± SE	15.4–16.5	14.6–16.0	15.8–17.0	15.8–17.1				
LH (U/L)					0.979	0.009	0.557	0.156
Mean	2.71	2.15	2.79	2.65				
Mean ± SD	1.57–4.16	1.28–3.26	1.67–4.20	1.64–3.90				
Adjusted mean	2.54	2.36	2.54	2.98				
Adjusted mean ± SE	2.38–2.70	2.16–2.57	2.38–2.71	2.78–3.19				
Inhibin B (pg/mL)					0.527	0.227	0.640	0.321
Mean	118.4	116.8	117.7	109.5				
Mean ± SD	74.8–172.0	78.9–162.2	74.3–171.1	56.8–179.2				
Adjusted mean	123.4	117.1	117.8	104.8				
Adjusted mean ± SE	116.0–131.1	107.7–126.9	110.5–125.3	96.9–113.0				

Abbreviations: CG, comparison group; EG, exposed group. Threshold for significance (α= 0.05) is 0.025.

a *p-*Values refer to differences adjusted by smoking status (total number of cigarettes per day during months of habitual smoking), chemical substances (mostly organic solvents, adhesives, paints, colors, wood, hides, or metal powders), age at the time of tests, BMI, alcohol use (g/day), education level, employment status, and abstinence time (days) for sperm data. Hormone data were not adjusted for education level, employment status, and abstinence time.

b Serum lipid basis.

c Ranges of serum TCDD concentrations in 1976 and 1998 are shown in Figure 2.

d Dioxin level in comparison groups was obtained from pools of sera from people living in uncontaminated areas.

e Values derived from back-transformation of log (sperm concentration, total sperm count, total motile sperm count, E_2_, testosterone, and FSH) and square-root transformation (semen volume, LH, and inhibin B); adjusted as described above. Normal values fall inside this range.

f Considered as A + B progressive motility of sperm, according to the WHO (1982).

g Considered as A + B progressive motility of sperm per total sperm count, according to the WHO (1982).
